# Targeted Delivery of Chemotherapy Agents Using a Liver Cancer-Specific Aptamer

**DOI:** 10.1371/journal.pone.0033434

**Published:** 2012-04-25

**Authors:** Ling Meng, Liu Yang, Xiangxuan Zhao, Lucy Zhang, Haizhen Zhu, Chen Liu, Weihong Tan

**Affiliations:** 1 Department of Chemistry and Department of Physiology and Functional Genomics, Shands Cancer Center and Center for Research at the Bio/Nano Interface, University of Florida, Gainesville, Florida, United States of America; 2 Department of Pathology, Immunology, and Laboratory Medicine, University of Florida College of Medicine, Gainesville, Florida, United States of America; 3 State Key Laboratory of Chemo/Bio-Sensing and Chemometrics, College of Biology, College of Chemistry and Chemical Engineering, Hunan University, Changsha, People’s Republic of China; Roswell Park Cancer Institute, United States of America

## Abstract

**Background:**

Using antibody/aptamer-drug conjugates can be a promising method for decreasing toxicity, while increasing the efficiency of chemotherapy.

**Methodology/Principal Findings:**

In this study, the antitumor agent Doxorubicin (Dox) was incorporated into the modified DNA aptamer TLS11a-GC, which specifically targets LH86, a human hepatocellular carcinoma cell line. Cell viability tests demonstrated that the TLS11a-GC-Dox conjugates exhibited both potency and target specificity. Importantly, intercalating Dox into the modified aptamer inhibited nonspecific uptake of membrane-permeable Dox to the non-target cell line. Since the conjugates are selective for cells that express higher amounts of target proteins, both criteria noted above are met, making TLS11a-GC-Dox conjugates potential candidates for targeted delivery to liver cancer cells.

**Conclusions/Significance:**

Considering the large number of available aptamers that have specific targets for a wide variety of cancer cells, this novel aptamer-drug intercalation method will have promising implications for chemotherapeutics in general.

## Introduction

It is well known that traditional cancer chemotherapy agents can cause serious side effects by their nonspecific toxicity. To overcome this problem and achieve specific drug delivery, our group and other investigators have used antibodies [1], [2] or aptamers [3], [4], [5], [6], [7], [8], [9], [10] to design ligand-linked drug conjugates for targeted-delivery applications.

Aptamers are single-stranded oligonucleotides which can specifically bind to small molecules, [11] peptides and proteins. [12] Aptamers not only provide the advantages of antibodies, such as high specificity and affinity, but they also have low immunogenicity and high stability, with easy synthesis and modification. Recently, a process called cell-SELEX (Systematic Evolution of Ligands by Exponential enrichment) has been developed to generate aptamers for specific recognition of target cancer cells, including T-cell acute lymphoblastic leukemia (T-cell ALL), small-cell lung cancers, liver cancers and virus-infected cells. [13], [14], [15], [16], [17], [18], [19] These aptamers are highly specific for different types of tumor cells and have excellent binding affinities. Because aptamers provide specificity at the molecular level, it is believed that aptamer-drug conjugates may enhance the efficiency of drug delivery, while at the same time, decreasing systemic toxicity.

Hepatocellular carcinoma (HCC) is one of the most common and deadly cancers in the world. It causes approximately 600,000 deaths every year. Currently, treatments for early liver cancer have relied on liver transplantation and surgical resection. Conventional chemotherapy has not been efficient with liver cancer patients, and since the chemotherapeutic agents are not specific to liver cancer cells, toxic side effects result. In a previous publication, we reported the development of a series of specific aptamers based on a mouse model. [14] One of these aptamers can also specifically recognize human liver cancer cells, and we report here a new design for the targeted delivery of Doxorubicin (Dox) to liver cancer cells.

Doxorubicin has been used for the treatment of liver cancer in the form of localized delivery, but its efficacy is impeded by toxic side effects. To overcome this problem, we have intercalated Dox into a modified aptamer probe. Dox is known to intercalate into the DNA strand by the presence of flat aromatic rings in the Dox molecule. Recent research has already shown that Doxorubicin can intercalate into aptamer A10 to provide specific killing efficiency to prostate cancer cells. [6], [20].

Aptamer TLS11a was previously selected by cell-SELEX against the BNL 1ME A.7R.1 (MEAR) mouse hepatoma cell line. [14] It was chosen for this application because it showed great binding affinity for LH86, a liver cancer cell line. [21] In order to achieve greater intercalation efficiency, a long GC tail was added to TLS11a to form a modified aptamer, TLS11a-GC. Through the interaction, the ratio between Doxorubicin and TLS11a-GC was 25∶1. Consequently, the delivery efficiency of Doxorubicin was much higher compared to the original TLS11a. Also, *in vitro* and *in vivo* experiments showed that TLS11a-GC-Dox conjugates have much better specific killing efficiency for target cancer cells compared to free Dox and control aptamer-Dox conjugates.

## Results

### The Binding Affinity of Aptamer TLS11a

**Figure 1:**
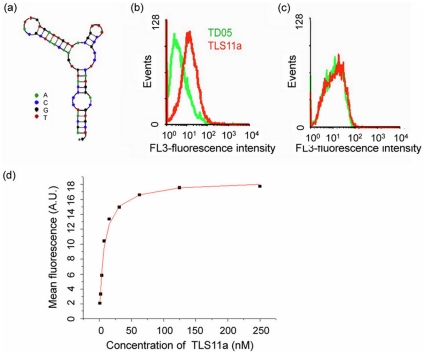
Characterization of aptamer TLS11a. (a) The secondary structure of aptamer TLS11a and its binding ability to (b) LH86 and (c) human normal liver cells, Hu1082. The green histogram shows the background binding (control aptamer, TD05), and the red fluorescence intensities show the binding of TLS11a with target and control cells. All probes were labeled with Phycoerythrin-Cy5.5. (d) Kd determination.

Aptamer TLS11a (Fig. 1a) was generated against the BNL 1ME A.7R.1 (MEAR) mouse hepatoma cell line [14] and showed strong binding affinity (Kd = 4.51±0.39 nM). [14] The LH86 cell line was established from a patient with liver cancer. [21] When TLS11a was used to test LH86 cells, obvious binding ability was observed (Fig. 1b). Also, when human normal liver cells, Hu1082, were tested using TLS11a, no significant binding was observed (Fig. 1c). In Fig. 1b and c, the green histogram shows the background binding (control aptamer, TD05), and the red fluorescence intensities show the binding of TLS11a with target and control cells. Compared to the control aptamer, there is a significant difference between the binding strength of TLS11a to LH86 and Hu1082 cells. No previously reported probe differentiates between liver cancer cells and human normal liver cells. Also, the Kd of TLS11a to LH86 was 7.16±0.59 nM (Fig. 1d), compared to 4.51±0.39 nM to BNL 1ME A.7R.1. [14].

Immunohistological imaging and fluorescence microscopy have been widely used in the study of solid tumors; therefore, we also assessed whether TLS11a could be used for tumor imaging with LH86, the positive cell line. Figure S1 shows the confocal images of LH86 detected with TLS11a and a control sequence, TD05 (Text S1). There was significant signal strength of TLS11a compared with the negative control, and the signal pattern shows that the aptamers bind to the surface of the cells.

It is usually assumed that aptamers selected against cell lines bind to cell membrane proteins. This has been demonstrated in most SELEX protocols involving tumor cell lines. [22], [23] In order to investigate the target molecule of TLS11a, we performed a protease assay, in which LH86 cells were treated with trypsin for 10 min at 37°C. After the incubation period, the protease activity was stopped with the addition of ice-cold PBS containing 20% FBS. The cells were quickly washed twice by centrifugation and then incubated with the aptamers. As shown in Figure S2, TLS11a lost recognition significantly in trypsin-treated cells (Text S1). The fluorescence signals reduced to the background, indicating that the treatment of the cells with the proteases caused digestion of the target protein, in turn showing that the target molecule of TLS11a is a membrane protein.

An internalization assay was then performed to determine if TLS11a could be internalized upon target binding. LH86 cells were first incubated with biotin- labeled TLS11a or TD05 and then further incubated with streptavidin- conjugated PE-Cy5.5. Then the buffer was removed, and culture medium with LysoSensor™ Green DND-189 was added to the cells and incubated at 37°C for two hours. LysoSensor served as an indicator of the lysosome location in the cells. As shown in Figure S3, there was clear internalization of the aptamer (Text S1). The TLS11a signal originated from inside the cells rather than on the outer margins, and it co-localized with LysoSensor. The control sequence showed no signal in either the 4°C or 37°C assays. The results suggest that TLS11a may have bound to a protein that could be internalized.

### Conjugation and Property Study of Aptamer-Dox Complex

**Figure 2:**
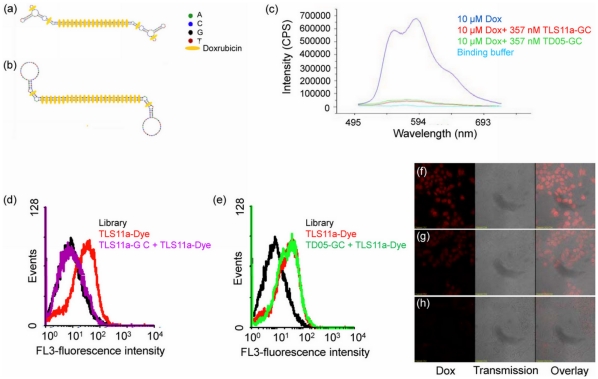
The secondary structure of aptamer-Dox conjugates and their recognition of target cells. The intercalation of Dox into GC-modified aptamers formed physical conjugates. (a) TLS11a-GC-Dox. (b) TD05-GC-Dox. (c) Quenching of Dox fluorescence after intercalation. The binding affinity of (d) TLS11a-GC or (e) TD05-GC to LH86 cells monitored using flow cytometry. The fluorescence signal is from Phycoerythrin-Cy5.5. Internalization of (f) Dox, (g) TLS11a-gc-Dox, and (h) TD05-GC-Dox observed by confocal microscopy.

Dox is known to intercalate within the DNA strand by the presence of flat aromatic rings, and it preferentially binds to double-stranded 5′-GC-3′ or 5′-CG-3′ sequences. [24], [25]. The secondary structure of TLS11a, as predicted by NUPACK software (http://www.nupack.org/), is shown in Figure 1a. According to the structure, there are two 5′-GC-3′ or 5′-CG-3′ sequences in the TLS11a sequence such that one TLS11a sequence can intercalate a maximum of two Doxorubicin molecules. In order to intercalate more Doxorubicin molecules, a long GC tail was added to the 5′ end of TLS11a to generate a modified aptamer, TLS11a-GC (Fig. 2a). Because of the long GC tail, TLS11a-GC forms a dimer structure. Nupack calculation indicated that one TLS11a-GC dimer could intercalate up to 56 Doxorubicin molecules to produce a TLS11a-GC-to-Doxorubicin ratio of 1∶28. A control aptamer sequence, TD05, was also modified with a long GC tail to give the same aptamer to Doxorubicin ratio (Fig. 2b). Even though the TLS1a-GC and TD05-GC dimers could intercalate up to 28 Dox per aptamer, the ratio between aptamer and Doxorubicin was kept at 1∶25 in these experiments.

It is well known that Dox has fluorescence properties, but the intercalation of Dox into DNA aptamer quenches the fluorescence of Dox because of the formation of charge-transfer complexes between the DNA bases and the anthracycline ring. [26], [27], [28] To examine whether such interaction occurs with modified TLS11a-GC and TD05-GC aptamers, fluorescence was acquired for Dox in the absence and in the presence of one of the aptamers (Fig. 2c). The solution of free Dox had the highest fluorescence signal compared to the black group (binding buffer). When modified aptamer (TLS11a-GC or TD05-GC) was added to the Dox solution at 1∶28 ratio and mixed well, the fluorescence dramatically decreased almost to the background level, indicating that the intercalation of Dox into DNA aptamer is feasible and rapid. Even after the aptamer-Dox complex solution was kept at room temperature for 3 h, the fluorescence stayed the same, indicating that the aptamer-Dox complex is very stable.

The binding affinity of TLS11a-GC was determined by a competition method. After incubating with unlabeled TLS11a-GC, the binding sites on LH86 cells were completely occupied. Then all cells were further incubated with dye-labeled TLS11a. Because all binding sites on the cell membrane were occupied by unlabeled TLS11a-GC, dye-labeled TLS11a could not bind to LH86 cells, and after washing no fluorescence signal was detected (Fig. 2d). At the same time, a competition experiment between TD05-GC and dye- labeled TLS11a was carried out. Because TD05-GC did not bind to LH86, the binding sites were available for interaction with dye-labeled TLS11a, resulting in a high fluorescence signal (Fig. 2e). After modification with the long GC tail, the flow cytometry data clearly showed that TLS11a-GC could still bind to LH86 cells, while TD05-GC could not.

Doxorubicin release was investigated using confocal microscopy. After 1 h incubation with Doxorubicin or aptamer-Dox conjugates, cells were further incubated for 3 h at 37°C before imaging. Figure 2f shows that cells treated with free Doxorubicin had the most Doxorubicin in their nuclei, while the nuclei of cells treated with TLS11a-GC-Dox conjugates (Fig. 2g) also contained Doxorubicin. However, the nuclei of cells treated with TD05-GC-Dox conjugates (Fig. 2h) contained almost no Doxorubicin. This experiment confirmed that TLS11a-GC-Dox conjugates had specific binding affinity to LH86 cells compared to TD05-GC-Dox conjugates. Furthermore, Dox could be released from TLS11a-GC-Dox conjugates after internalization and could enter the nucleus.

### Cell Toxicity of Aptamer-Dox Conjugates

**Figure 3:**
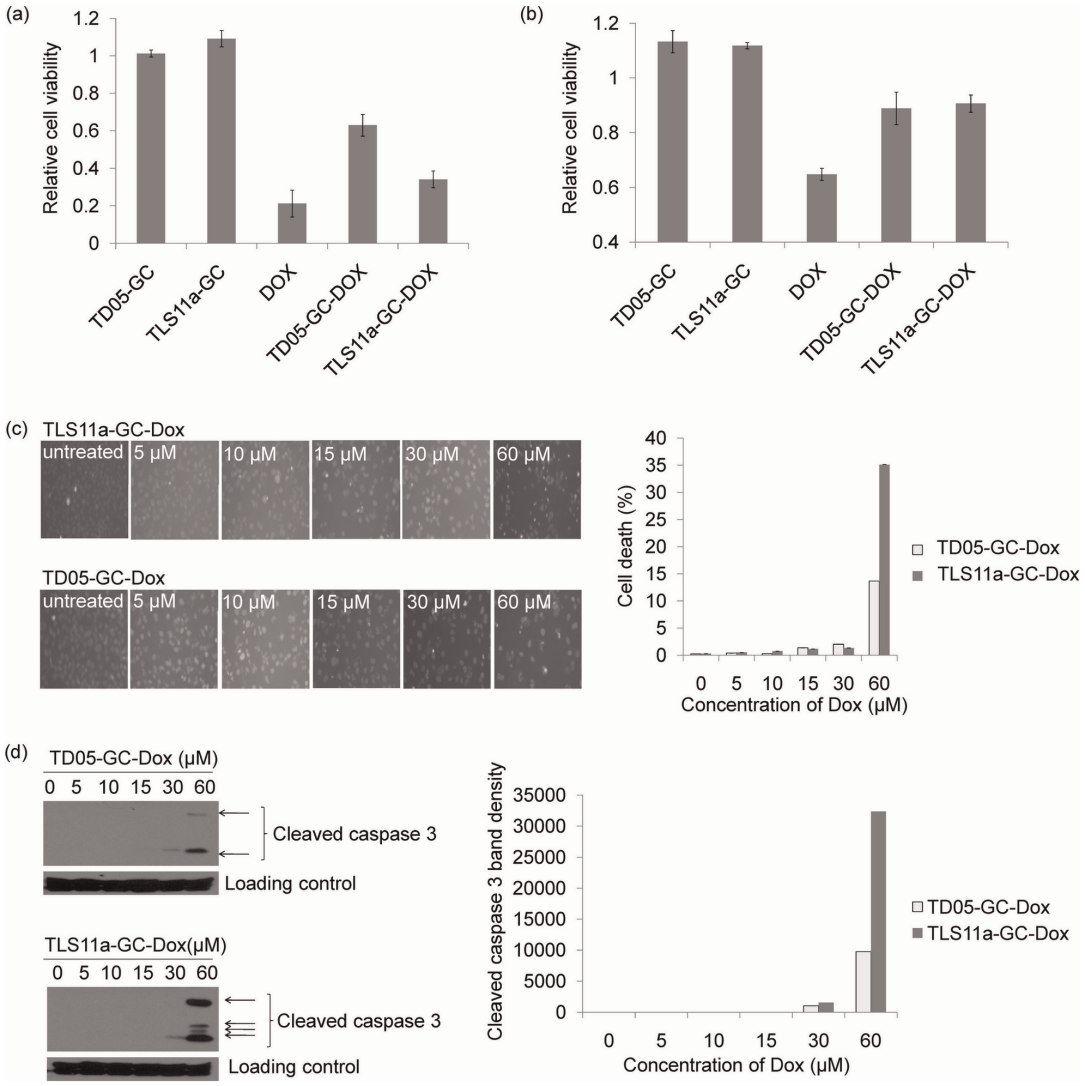
Relative cell viability and apoptosis of cells treated with TLS11a-GC, TD05-GC, free Doxorubicin, TLS11a-GC-Dox or TD05-GC-Dox. (a) Relative cell viability of LH86 (target cell line); (b) Relative cell viability of Hu1229 (human normal liver cells); (c) Hoechst 33258 staining for apoptotic and dead LH86 cells treated with a series of concentrations of TLS11a-GC-Dox or TD05-GC-Dox; (d) Western blot results for cleaved caspase 3 in LH86 cells treated with a series of concentrations of TLS11a-GC-Dox or TD05-GC-Dox.

The cell viability of LH86 treated with TLS11a-GC-Dox, TD05-GC-Dox, Doxorubicin, TLS11a-GC, or TD05-GC was tested and compared to that of untreated cells (Fig. 3a). The Doxorubicin concentration in TLS11a-GC-Dox, TD05-GC-Dox, and free Doxorubicin was kept at 7.5 µM, and the ratio of Doxorubicin to aptamer was 25∶1. The aptamer concentration in the TLS11a-GC, TD05-GC, TLS11a-GC-Dox, and TD05-GC-Dox groups was kept at 300 nM. From the data shown in Figure 3a, TLS11a-GC and TD05-GC had no significant effect on cell viability. It is obvious that treatment with TLS11a-GC-Dox conjugates decreased cell viability. The efficiency of cell toxicity was Doxorubicin >TLS11a-GC-Dox>TD05-GC-Dox. Even though the cell toxicity of TLS11a-GC-Dox was less than that of free Doxorubicin, the toxicity effect of TD05-GC-Dox was much poorer. Further experiments using Hu1229 human normal liver cells (Fig. 3b) showed that free Dox had significant toxicity, while TLS11a-GC-Dox and TD05-GC-Dox had similar and very limited toxicity. These data demonstrate that the specificity of aptamer TLS11a-GC to LH86 cells achieved toxicity to target cells only.

Cell apoptosis was investigated using Hoechst 33258 staining (Fig. 3c). From the fluorescence images, when the Dox concentration was 60 µM, there were more apoptotic and dead cells in the TLS11a-GC-Dox group (35.1%) than in the TD05-GC-Dox group (13.7%), further indicating the specificity of aptamer-Dox conjugates. In addition, caspase 3 activation was monitored using Western blot (Fig. 3d). Caspase 3 plays an important role in cell apoptosis, and cleaved caspase 3 indicates its activation. From the data shown, when the Dox concentration was 60 µM, the band density of cleaved caspase 3 in cells treated with TLS11a-GC-Dox was 3.3 times higher than that in cells treated with TD05-GC-Dox.

### *In Vivo* Studies

**Figure 4:**
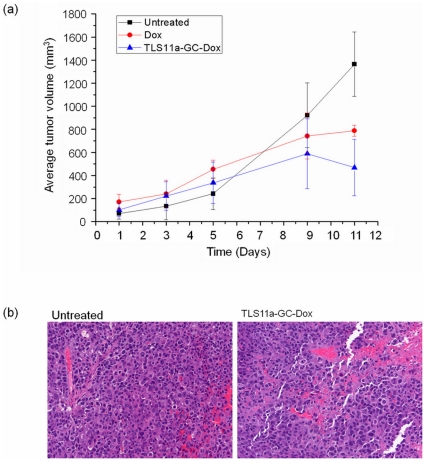
Tumor inhibition of the aptamer-Dox complex in mouse model. (a) Average tumor volume of mice treated with nothing (black), Doxorubicin (red), or TLS11a-GC-Dox (blue); (b) Microscopy images of H&E stained tumor tissue slides.

Thirty NOD. Cg-Prikdc (scid) IL2 mice were treated as described in the experimental section. The tumor size of each mouse was measured every other day, and the average tumor volume was calculated (Fig. 4a). The data show that both free Dox and TLS11a-GC-DOX complex had obvious tumor inhibition effects compared to the untreated group. Also, the TLS11a-GC-DOX complex had a more efficient effect compared to free Dox, indicating that TLS11a-GC-Dox conjugates targeted the tumor cells and achieved higher local Dox concentration in the tumor site compared to free Dox. Also, tumors of each mouse were collected and fixed using 10% formalin for 24 h. Then all samples were sent to the molecular pathology core lab for H&E staining. From the H&E stained slides, the TLS11a-GC-DOX complex-treated tumor (Fig. 4b, right) showed significant tumor cell necrosis compared to the untreated tumor (Fig. 4b, left).

## Discussion

Aptamer TLS11a was generated using mouse liver cancer cells, but it also shows high binding affinity to human liver cancer cells. Furthermore, TLS11a is the first aptamer to be identified as specific for human liver cancer cells. Our results showed that the target molecule of TLS11a is very likely a membrane protein which can be internalized into cells. These results indicate that TLS11a may be a useful aptamer for targeted drug delivery in liver cancer treatment.

Doxorubicin plays a very important role in liver cancer treatment, and it is considered to be the most utilized anticancer drug worldwide. Dox is able to inhibit cell proliferation through intercalation of DNA in the cell’s nucleus. Several reports have demonstrated that free Dox is membrane-permeable and can be uptaken by cells through a passive diffusion mechanism, rapidly transported to the nuclei, and bound to the chromosomal DNA. [29] Therefore, while Dox is generally toxic to proliferating cells, it is also toxic to normal cells, thus limiting its therapeutic efficacy in clinical use. Here, by making use of modification of a specific liver cancer aptamer and the intercalation property of Dox, we generated an easy, rapid, and efficient method to deliver Dox to targeted cancer cells. During *in vitro* experiments, we proved the specific binding affinity of modified TLS11a-GC to target LH86 cells, but non-recognition to human normal liver cells. Furthermore, we demonstrated the specific toxicity of TLS11a-GC-Dox complex to target cells, compared to normal liver cells, thereby limiting its toxicity to only target cells. This targeting was achieved through the specific binding affinity of aptamer TLS11a. Although TLS11a-GC-Dox achieved good cell toxicity compared to control TD05-GC-Dox during MTS assay, less toxicity was observed in the TLS11a-GC-Dox group than in the free Dox group. Also, less internalization and release of Dox in the TLS11a-GC-Dox group than in the free Dox group was observed using confocal microscopy. Dox itself is a small molecule which can be rapidly uptaken by cells through a passive diffusion mechanism. Within 15 min, cells treated with free Dox show an intense red fluorescence in the nuclear region, indicating that the uptake speed is very rapid. [29] However, once Dox was intercalated into DNA aptamer to form a much larger molecule, the cell uptake of aptamer-Dox complex was mainly dependent on the aptamer and its cell membrane target. In addition, aptamer internalization required more time (about 2 h) than free Dox, thus slowing the internalization of aptamer-Dox complex and the release of Dox from the complex. Therefore, less toxicity was observed in the TLS11a-GC-Dox group than in the free Dox group during *in vitro* experiments. During *in vivo* experiments, TLS11a-GC-Dox had decreased cell internalization speed compared to free Dox. However, because of target recognition by TLS11a aptamer, the local concentration of Dox was increased, compared to free Dox. Hence, higher tumor inhibition efficacy was achieved in the TLS11a-GC-Dox-treated mouse group.

In summary, by making use of the ability of anthracycline drugs to intercalate between bases of nucleotides, a new design to modify aptamer TLS11a to TLS11a-GC and make Dox and TLS11a-GC conjugates was investigated. The specificity and efficacy of this conjugate to serve as a drug-delivery platform was further demonstrated *in vitro* and *in vivo*. Our data showed that the modified aptamer retains its specificity and can load much more Dox than the unmodified aptamer. Also, the aptamer-Dox conjugates are stable in cell culture medium and can differentially target LH86 cells. The specificity of this system was further demonstrated by treatment of human normal liver cells, which lack the aptamer binding target. The aptamer-Dox conjugate prevents the nonspecific uptake of Dox and decreases cellular toxicity to the non-target cells. Furthermore, the *in vivo* experiment showed better tumor inhibition by the TLS11a-GC-Dox group compared to all other control groups, indicating the successful delivery of Dox by the modified aptamer. This targeting specificity assured a higher local Dox concentration in the tumor. In addition, aptamer-Dox conjugates are smaller than antibody-based drug delivery systemsand some other delivery system, [30], [31] allowing faster penetration and fewer immunoreactions. We anticipate that the newly designed aptamer-Dox conjugation platform, which is based on the intercalation of anthracyclines within the bases of aptamers, may be utilized in distinct ways to develop novel targeted therapeutic modalities for more effective cancer chemotherapy.

## Materials and Methods

### Cell Culture and Reagents

LH86 cells have been described previously. [21] Cells were cultured in Dulbecco’s modified Eagle medium (DMEM) supplemented with 10% fetal bovine serum (FBS) (heat inactivated) and 100 IU/mL penicillin-streptomycin at 37°C in a humid atmosphere with 5% CO_2_. Doxorubicin hydrochloride (Dox) was purchased from Fisher Scientific (Houston, TX, USA). All reagents for DNA synthesis were purchased from Glen Research. Unless otherwise noted, reactants, buffers and solvents were obtained commercially from Fisher Scientific.

### Conjugation of Aptamer-Dox

Aptamer TLS11a (5′- *ACAGCATCCCCATGTGAACAATCGCATTGTGATTG TTACGGTTTCCGCCTCATGGACGTGCTG* -3′); a control sequence TD05.

(5′-CACCGGGAGGATAGTTCGGTGGCTGTTCAGGGTCTCCTCCCG GTG-3′); modified aptamer TLS11A-GC (5′- *CGCGCGCGCGCGCGC GCGCGCGCGCGCGCGCGCGCGCGCGCGCGCGCGCGCGCGCGCGACAGCATCCCCATGTGAACAATCGCATTGTGATTGTTACGGTTTCCGCCTCATGGACGTGCT G* -3′); and modified control sequence TD05-GC (5′- *CGCGCGCGCGCGCGCGCGCGCGCGCGCGCGCGCGCGCGCGCGCGCGCGCGCGCGCGCGCGAACACCGTGGAGGATAGTTCGGTGGCTGTTCAGGGTCTCCTCCCGGTG* -3′) were synthesized on an ABI3400 DNA/RNA synthesizer (Applied Biosystems, Foster City, CA, USA). The completed sequences were then deprotected in AMA (ammonium hydroxide/40% aqueous methylamine 1∶1) at 65°C for 30 min and further purified by reversed-phase HPLC (ProStar, Varian, Walnut Creek, CA, USA) on a C-18 column using 100 mM trimethylamine acetate buffer (TEAA, pH7.5) and acetonitrile as the mobile phase. To make aptamer-Dox conjugates, either TLS11a-GC or TD05-GC was mixed with Doxorubicin in binding buffer (PBS containing 5 mM MgCl_2,_ 4.5 mg/mL glucose, 0.1 mg/mL yeast tRNA, 1 mg/mL BSA) or DMEM media at a 1∶25 ratio of aptamer to Dox.

### Monitoring of Complex Formation by Fluorescence

Physical conjugates between aptamer (TLS11a or TD05) and Doxorubicin were prepared at a 1∶28 molar ratio of aptamer to Doxorubicin (10 µM) in binding buffer, and fluorescence was monitored at 500–720 nm (1.5 mm slit) on a Fluorolog-Tau-3 Spectrofluorometer (Jobin Yvon) with excitation at 480 nm.

### Determination of Aptamer Affinity

The LH86 cells were detached from dishes using nonenzymatic cell dissociation solution (Cellgro) and then washed with washing buffer (PBS containing 5 mM MgCl_2_ and 4.5 mg/mL glucose). The binding affinity of TLS11a was determined by incubating LH86 cells (10^5^) on ice for 30 min with a series of concentrations of biotin-labeled TLS11a in binding buffer (100 µL). Cells were then washed twice with washing buffer (1.0 mL) and suspended in fluorescein-labeled streptavidin (0.1 mL) for further incubation (15 min on ice). Before flow cytometric analysis, cells were washed with washing buffer twice and suspended in washing buffer (0.2 mL). The mean fluorescence intensity of cells was used to calculate the equilibrium dissociation constant (Kd) of the TLS11a and LH86 cell interaction by fitting the dependence of fluorescence intensity (F) on the concentration of the biotin-labeled TLS11a (L) to the equation F = Bmax[L]/(Kd+[L]). The binding assay experiments were repeated at least three times.

To monitor the binding affinity of TLS11-GC, a competition experiment was carried out. Briefly, 200 nM TLS11a-GC was incubated with LH86 cells for 20 min on ice, and then 1 µM biotin-labeled TLS11a was added for 15 min further incubation. Cells were then washed twice with washing buffer (1.0 mL) and suspended in fluorescein-labeled streptavidin (0.1 mL) for further incubation (15 min on ice). Before flow cytometric analysis, cells were washed with washing buffer twice and suspended in washing buffer (0.2 mL).

### Assessment of Cell Uptake of Dox by Confocal Microscopy

For confocal imaging, Doxorubicin or aptamer-Dox conjugates were incubated with a LH86 cell monolayer in a 35-mm glass bottom culture dish (Mat Tek Corp.) in DMEM media at 37°C for 1 h. After washing once using media, fresh media were added to dishes for further 3 h incubation at 37°C. Then the dishes with cells were placed above a 40×objective of an Olympus FV500-IX81 confocal microscope (Olympus America Inc., Melville, NY). A 5-mW, 488-nm Ar+ laser was then used for excitation of Doxorubicin. The objective used for imaging was an Olympus LC Plan F1 40X/0.60 ph 2 40× objective.

### MTS Cell Viability Assay

Chemosensitivity of LH86 to Dox or aptamer-Dox conjugates was determined using the CellTiter 96 cell proliferation assay (Promega, Madison, WI, USA). Briefly, a 100 µL aliquot of LH86 cells (5×10^4^ cells/mL) was seeded in 96-well plates (*n* = 3) and allowed to grow overnight. Afterwards, cells were treated with 100 µL of: 1) control aptamer TD05-GC, 2) aptamer TLS11a-GC, 3) Dox, 4) TD05-GC-Dox physical conjugate (25∶1 Doxorubicin to TD05-GC mole ratio), or 5) TLS11a-GC-Dox physical conjugate (25∶1 Doxorubicin to TLS11a mole ratio) for 1 hour, washed, and further incubated in fresh media for a total of 48 hrs. For cytotoxicity measurement, media were removed from each well, and then CellTiter reagent (20 µL) and media (100 µL) were added to each well and incubated for 3 h. Using a plate reader (Tecan Safire microplate reader, AG, Switzerland), the absorption was recorded at 490 nm. The percentage of cell viability was determined by comparing Dox and aptamer-Dox conjugate-treated cells with the untreated control.

### Hoechst 33258 Staining for Apoptotic Cells

Cell apoptosis was determined by nucleus morphology change. LH86 cells in exponential growth were placed in a 48-well plate at a final concentration of 1.5×10^4^ cells per well. After 12 h, cells were treated with different concentrations of TD05-GC-Dox physical conjugate (25∶1 Doxorubicin to TD05-GC mole ratio) or TLS11a-GC-Dox physical conjugate (25∶1 Doxorubicin to TLS11a mole ratio) for 1 hour, washed, and further incubated in fresh media for a total of 48 hrs. Subsequently, cells were washed twice with PBS and stained with Hoechst 33258 staining solution according to the manufacturer’s instructions. After incubation at 37°C for 10 min, cell nucleus fragmentation/condensation was detected by fluorescence microscopy. Apoptotic cell death was assessed by calculating the number of apoptotic cells with condensed nuclei in six to eight randomly selected areas. The results shown represent three independent experiments.

### Western Blotting Analysis

Cells were harvested and washed twice with phosphate saline buffer. The cell pellets were resuspended in lysis buffer containing Nonidet P-40 (10 mM HEPES, pH 7.4, 2 mM EGTA, 0.5% Nonidet P-40, 1 mM NaF, 1 mM NaVO_4_, 1 mM phenylmethylsulfonyl fluoride, 1 mM dithiothreitol, 50 µg/ml trypsin inhibitor, 10 µg/ml aprotinin, and leupeptin) and incubated on ice for 30 min. After centrifugation at 12000×g at 4°C for 15 min, the supernatant was transferred to a fresh tube, and the protein concentration was determined. Equivalent samples (20 µg of protein) were subjected to SDS-PAGE on 12% gels. The proteins were transferred onto nitrocellulose membranes and probed with the indicated primary antibodies (cleaved caspase-3 antibody, Cell Signaling Technology, Inc.), followed by the appropriate secondary antibodies conjugated with horseradish peroxidase (HRP, Santa Cruz Biotechnology, Inc.). Immunoreactive bands were detected using enhanced chemiluminescence (ECL, Pierce). The molecular sizes of the proteins detected were determined by comparison with prestained protein markers (Bio-Rad). All band densities were calculated using ImageJ software.

### *In vivo* Experiment

The NOD. Cg-Prkdc (scid) IL2 mice were purchased from Jackson Laboratory (Bar Harbor, ME) and housed in the animal facility at the University of Florida with institutional regulatory approval (Institutional Animal Care and Use Committee). This study was approved by Institutional Animal Care and Use Committee with approval ID IC00001331. Thirty NOD. Cg-Prkdc (scid) IL2 mice were subcutaneously injected with 7×10^6^ *in vitro*-propagated LH86 cells. When the tumors could be easily seen and measured, mice were divided into three groups: (1) group 1, untreated; (2) group 2, treated with free Dox; and (3) group 3, treated with TLS11a-GC-DOX complex. The Doxorubicin dosage was kept the same in groups 2 and 3 at 2 mg/kg. All treatments continued for 11 days. Drugs were injected through tail vein on days 1, 2, 3, 4, 5, 9, 10, and all samples were collected on day 11. The tumor size for each mouse was measured every other day. The heart, lung, liver, kidney and tumor of each mouse were collected on day 11 and fixed using 10% formalin for 24 h at room temperature, and then hematoxylin and eosin staining (H&E staining) was carried out.

## Supporting Information

Figure S1 **Confocal images of aptamer staining with cultured LH86 cells.** Cells were incubated with aptamer conjugated with biotin, and the binding event was observed with AlexaFluor 633-conjugated streptavidin. Non-binding sequence TD05 showed the background binding. Aptamers show significant binding over the background signal.
(TIF)

Figure S2 **Preliminary determination of the type of cell-surface molecule which binds to TLS11a.** Cells were treated with trypsin for 10 min and then incubated with aptamer.
(TIF)

Figure S3 **Co-localization of (a) TLS11a or (b) control TD05 and Lysosensor in endosomes after two hours of incubation at 37°C.**
(TIF)

Text S1 **Supporting methods.**
(DOC)
