# Supplementary figures and images for: Targeted Delivery of Chemotherapy Agents Using a Liver Cancer-Specific Aptamer
Source: PLoS One. 2012 Apr 25;7(4):e33434. doi: 10.1371/journal.pone.0033434 (PMC3338807; doi:10.1371/journal.pone.0033434)

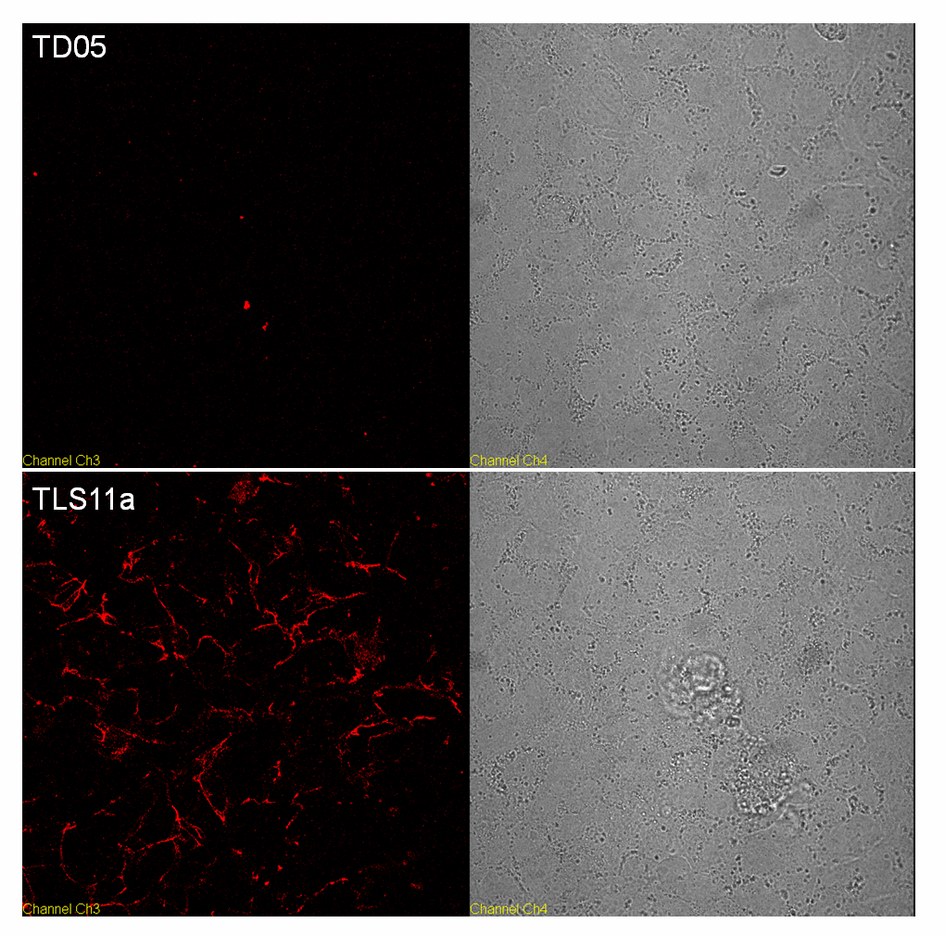

Supplement: Figure S1 — Confocal images of aptamer staining with cultured LH86 cells. Cells were incubated with aptamer conjugated with biotin, and the binding event was observed with AlexaFluor 633-conjugated streptavidin. Non-binding sequence TD05 showed the background binding. Aptamers show significant binding over the background signal. (TIF) [file pone.0033434.s001.tif]

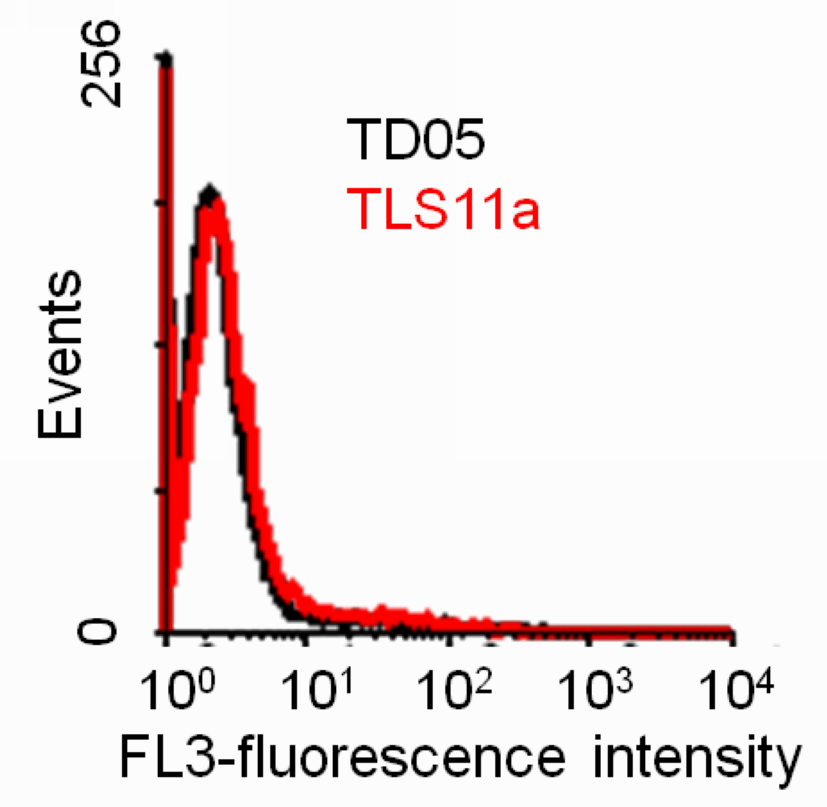

Supplement: Figure S2 — Preliminary determination of the type of cell-surface molecule which binds to TLS11a. Cells were treated with trypsin for 10 min and then incubated with aptamer. (TIF) [file pone.0033434.s002.tif]

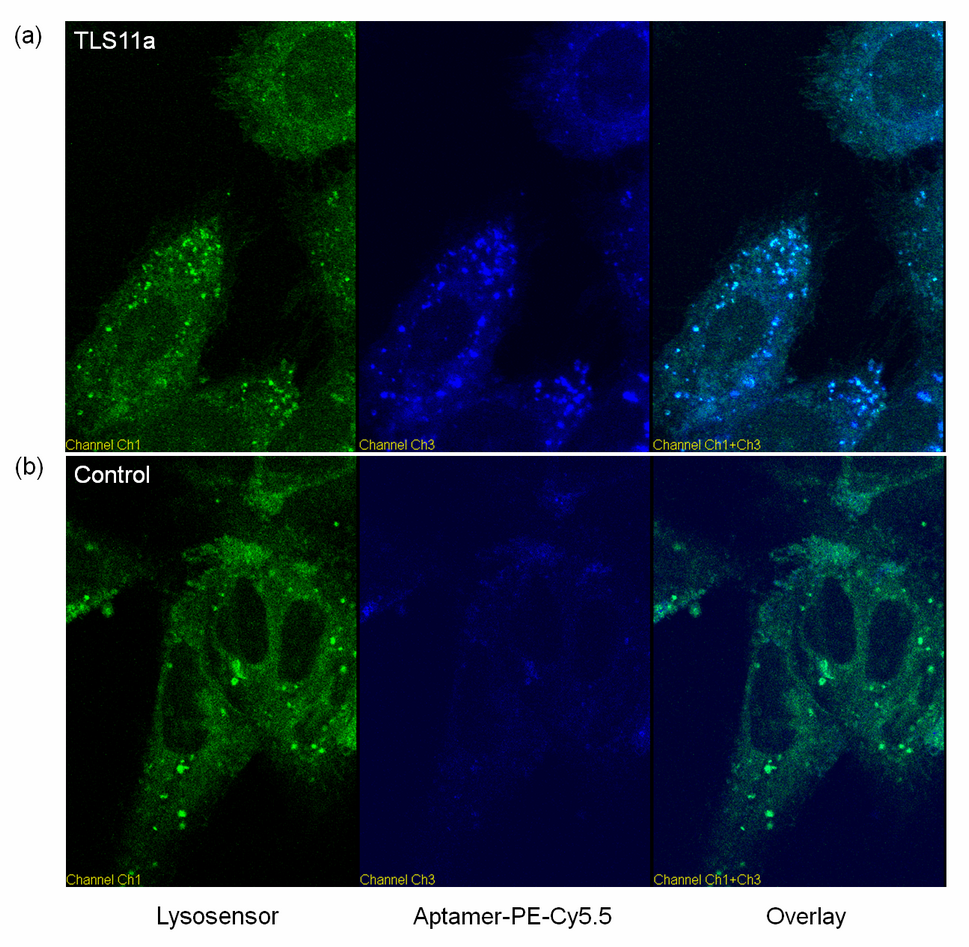

Supplement: Figure S3 — Co-localization of (a) TLS11a or (b) control TD05 and Lysosensor in endosomes after two hours of incubation at 37°C. (TIF) [file pone.0033434.s003.tif]
