# Supplementary material for: Targeted Delivery of Chemotherapy Agents Using a Liver Cancer-Specific Aptamer
Source: PLoS One. 2012 Apr 25;7(4):e33434. doi: 10.1371/journal.pone.0033434 (PMC3338807; doi:10.1371/journal.pone.0033434)
Supplement: Text S1 — Supporting methods. (DOC) [file pone.0033434.s004.doc]

**Targeted delivery of chemotherapy agents using a liver cancer-specific aptamer**

*Ling Meng^1^, Liu Yang^1^ , Xiangxuan Zhao^1^ , Lucy Zhang^1^ , Haizhen Zhu^2^ , Chen Liu^1^* , Weihong Tan^1^**

1. Department of Chemistry and Department of Physiology and Functional Genomics, Shands Cancer Center and Center for Research at the Bio/nano Interface, University of Florida, Gainesville, FL 32611-7200; Department of Pathology, Immunology, and Laboratory Medicine, University of Florida College of Medicine, Gainesville, FL 32610
2. State Key Laboratory of Chemo/Bio-Sensing and Chemometrics, College of Biology, College of Chemistry and Chemical Engineering, Hunan University, Changsha, 410082, P. R. China.

**Supporting information**

Confocal Microscopy of Cultured Cells

The binding of TLS11a with LH86 cells was further assessed by confocal microscopy. Here, LH86 cells were seeded in a 35-mm Petri dish, 10 mm microwell (MatTek Corporation), and cultured overnight. The cells showing more than 60% confluence were carefully washed and then incubated with the biotin-labeled TLS11a or control TD05 at a final concentration of 200 nM. After incubation at 4ºC for 30 min, cells were carefully washed before further incubation with a 1:200 dilution (optimized) of streptavidin-conjugated AlexaFluor 633 (Invitrogen) for 15 min. Excess probes were removed by washing, and the signal was detected by confocal microscopy (FV500-IX81 confocal microscope, Olympus America Inc., Melville, NY) using a 40x oil immersion objective (NA=1.40, Olympus, Melville, NY). A 633 nm laser line was used for excitation, and the emitted light was passed through a LP650 filter prior to detection.

Protease Assay

Extracellular membrane proteins are common targets of cell-SELEX, as demonstrated by many cell-SELEX schemes. In this work, protease assays were performed to determine surface molecules to which the aptamers bind. LH86 cells were dissociated with nonenzymatic dissociation solution. The cells were washed twice with washing buffer and incubated with trypsin (Cellgro) solution (0.05% trypsin/0.53 mM EDTA in HBSS) for 10 min at 37ºC. After incubation, ice-cold washing buffer containing 20% FBS was added to halt the protease activity. Cells were quickly centrifuged at 1000 rpm for 5 min and washed twice with washing buffer. The cell pellets were incubated with aptamers in a binding buffer, and the signal was then detected by flow cytometry.

**Internalization study**

A co-localization experiment was carried out. As described above, LH86 cells were first incubated with biotin-labeled TLS11a or TD05 and then further incubated with a 1:400 dilution (optimized) of streptavidin-conjugated PE-Cy5.5 (Invitrogen). After washing, DMEM media with 1:1000 dilution of LysoSensor™ Green DND-189 (Invitrogen) was added to the dishes. After incubation at 37ºC for 2 h, cells were washed twice with washing buffer, and the fluorescence signal was detected by confocal microscopy. A 488 nm laser line was used for excitation, and the emitted light was passed through a LP650 filter prior to detection.
